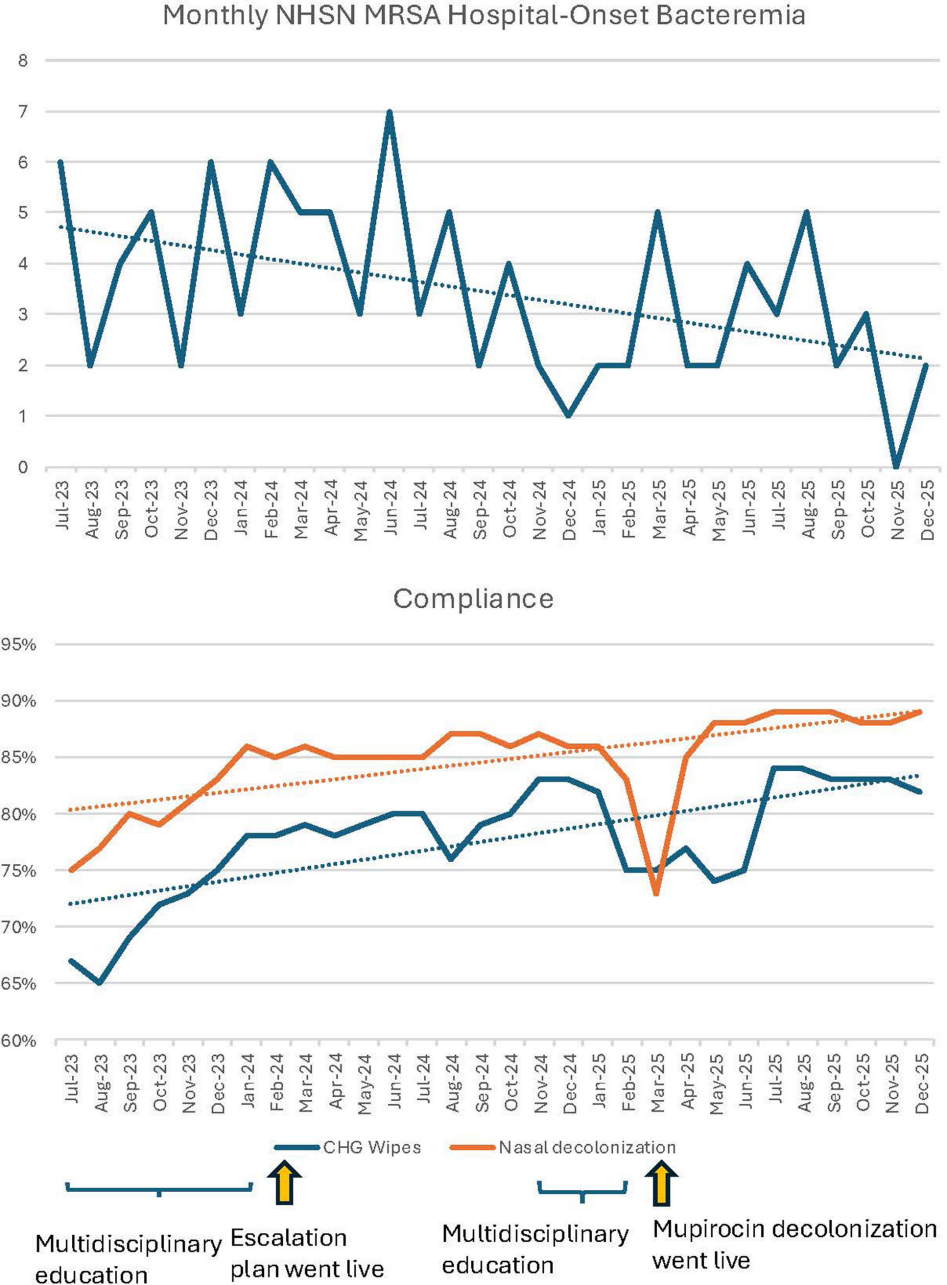# 36 Clean Hands, Safe Patients: Continuing a Culture Focused on Hand Hygiene

**DOI:** 10.1017/ash.2026.10477

**Published:** 2026-06-23

**Authors:** Nicholas Van Sickels, Takaaki Kobayashi, Rachel Howard, Amanda Green, Kimberly Blanton, Sarah Cecil, Julian Steele, Yosef Wirian, Rahul Agrawal

**Affiliations:** 1 University of Kentucky; 2 UK Healthcare; 3 UK HealthCare; 4 University of Kentucky Healthcare

## Abstract

**Background:** Following the COVID-19 pandemic, the University of Kentucky (UKY) experienced elevated rates of methicillin-resistant Staphylococcus aureus (MRSA). To drive rapid improvement, an MRSA escalation program was developed incorporating targeted contact precautions for units not meeting decolonization thresholds, enhanced MRSA surveillance testing, and data-driven reports to support unit leadership. **Methods:** The UKY Infection Prevention and Control Program (IPAC) implemented a data-driven MRSA escalation strategy. At baseline, all patients in intensive care units, patients with indwelling lines or tubes, and patients testing positive for MRSA were expected to undergo daily chlorhexidine bathing and intranasal povidone iodine decolonization. Upon review, gaps were identified in decolonization performance and screening of at-risk populations. The intervention addressed these gaps by including electronic health record (EHR)–prompted MRSA screening for at-risk patients, development of aggregate decolonization compliance dashboards, and real-time performance dashboards for unit supervisors. A monthly decolonization performance threshold of 70 percent was established. Units with persistent compliance below 70 percent were placed on contact precautions with an “MRSA Escalation” designation in the EHR. IPAC met with identified units to provide toolkits and education on dashboard utilization and integration of MRSA metrics into routine huddles and sprints. In March 2025, intranasal decolonization was transitioned from povidone iodine to mupirocin. Monthly hospital-onset MRSA bacteremia counts, chlorhexidine bathing compliance, and nasal decolonization compliance from July 2023 through December 2025 were analyzed using simple linear regression with time in months as the independent variable. **Results:** Multidisciplinary education was conducted across nursing, physician, and leadership groups from August 2023 through January 2024, with program go-live in February 2024. Chlorhexidine bathing compliance increased significantly over time, with an average improvement of 0.39 percentage points per month (95% CI 0.24–0.55, p < 0.001, R² = 0.48, Figure 1). Nasal decolonization compliance also increased significantly, with an average improvement of 0.30 percentage points per month (95% CI 0.16–0.44, p < 0.001, R² = 0.40). Hospital-onset MRSA bacteremia demonstrated a significant downward trend (slope –0.089 cases per month, p = 0.012, R² = 0.20), corresponding to an average reduction of approximately one case every 11 months. Quarterly SIR decreased from 1.10 in Q3 2023 to 0.54 in Q4 2025. **Conclusions:** Implementation of an MRSA escalation program incorporating decolonization performance thresholds, targeted contact precautions, enhanced education, real-time data reporting, and transition to mupirocin for nasal decolonization was associated with significant improvements in decolonization compliance and a sustained reduction in hospital-onset MRSA bacteremia.

**Figure:**